# Evolutionary relationships of Aurora kinases: Implications for model organism studies and the development of anti-cancer drugs

**DOI:** 10.1186/1471-2148-4-39

**Published:** 2004-10-12

**Authors:** James R Brown, Kristin K Koretke, Marian L Birkeland, Philippe Sanseau, Denis R Patrick

**Affiliations:** 1 Bioinformatics Division, Genetics Research, GlaxoSmithKline, 1250 South Collegeville Road, UP1345, P.O. Box 5089, Collegeville Pennsylvania 19426-0989, USA; 2 Dept. of Molecular Oncology, Microbial, Musculoskeletal and Proliferative Disease Center for Excellence in Drug Discovery, GlaxoSmithKline, 1250 South Collegeville Road, UP1345, P.O. Box 5089, Collegeville Pennsylvania 19426-0989, USA

## Abstract

**Background:**

As key regulators of mitotic chromosome segregation, the Aurora family of serine/threonine kinases play an important role in cell division. Abnormalities in Aurora kinases have been strongly linked with cancer, which has lead to the recent development of new classes of anti-cancer drugs that specifically target the ATP-binding domain of these kinases. From an evolutionary perspective, the species distribution of the Aurora kinase family is complex. Mammals uniquely have three Aurora kinases, Aurora-A, Aurora-B, and Aurora-C, while for other metazoans, including the frog, fruitfly and nematode, only Aurora-A and Aurora-B kinases are known. The fungi have a single Aurora-like homolog. Based on the tacit assumption of orthology to human counterparts, model organism studies have been central to the functional characterization of Aurora kinases. However, the ortholog and paralog relationships of these kinases across various species have not been rigorously examined. Here, we present comprehensive evolutionary analyses of the Aurora kinase family.

**Results:**

Phylogenetic trees suggest that all three vertebrate Auroras evolved from a single urochordate ancestor. Specifically, Aurora-A is an orthologous lineage in cold-blooded vertebrates and mammals, while structurally similar Aurora-B and Aurora-C evolved more recently in mammals from a duplication of an ancestral Aurora-B/C gene found in cold-blooded vertebrates. All so-called Aurora-A and Aurora-B kinases of non-chordates are ancestral to the clade of chordate Auroras and, therefore, are not strictly orthologous to vertebrate counterparts. Comparisons of human Aurora-B and Aurora-C sequences to the resolved 3D structure of human Aurora-A lends further support to the evolutionary scenario that vertebrate Aurora-B and Aurora-C are closely related paralogs. Of the 26 residues lining the ATP-binding active site, only three were variant and all were specific to Aurora-A.

**Conclusions:**

In this study, we found that invertebrate Aurora-A and Aurora-B kinases are highly divergent protein families from their chordate counterparts. Furthermore, while the Aurora-A family is ubiquitous among all vertebrates, the Aurora-B and Aurora-C families in humans arose from a gene duplication event in mammals. These findings show the importance of understanding evolutionary relationships in the interpretation and transference of knowledge from studies of model organism systems to human cellular biology. In addition, given the important role of Aurora kinases in cancer, evolutionary analysis and comparisons of ATP-binding domains suggest a rationale for designing dual action anti-tumor drugs that inhibit both Aurora-B and Aurora-C kinases.

## Background

The Auroras are a conserved family of serine/threonine kinases which have essential functions in cell division [1,2]. In mitosis, Aurora kinases are required for chromosome segregation, condensation and orientation in the metaphase plate, spindle assembly, and the completion of cytokinesis.

Model organism studies have played a pivotal role in functional characterization of Aurora kinases. Aurora kinases were first identified as mutant alleles in *Drosophila melanogaster *(fruitfly) that caused defective spindle-pole formation [3]. Subsequently, *Drosophila *was found to have a second Aurora homolog [4], and the nematode, *Caenorhabditis elegans*, similarly has two Aurora-like genes [5,6]. The fungi, *Saccharomyces cerevisiae *and *Schizosaccharomyces pombe*, have a single Aurora, known as increase-in-ploidy 1 (Ipl1) [7] and Aurora-related kinase 1 (Ark1) [8], respectively. Among cold-blooded vertebrates, Aurora kinases have been most widely studied in the frog, *Xenopus laevis*, which has two kinases; Aurora-A and Aurora-B [9,10]. More recently discovered is a third Aurora kinase called Aurora-C in rodents and humans [11].

The Aurora kinases are mitotic kinases that generally associate with chromosomes, often in complexes with other proteins, and interact with cytoskeletal components in cell division. The three mammalian Aurora kinases appear at specific locations during mitosis. Aurora-A, the "polar kinase", primarily associates with the separating centrosomes while Aurora-B, the "equatorial kinase", is a chromosomal passenger protein [1]. The least studied Aurora kinase, Aurora-C, appears to be localized to the centrosome from anaphase to telophase and is highly expressed in the testis [11,12].

Recent studies indicate that all three Aurora kinases have strong associations with cancer. Aurora-A has been mapped to a region in the human chromosome (20q13.2-13.3) that is amplified in cancer cell lines and primary tumors [13,14]. Transfected mouse cell lines with Aurora-A have been shown to cause tumors when injected into nude mice [14,15] and a polymorphic variant (amino acid substitution Phe31Ile) has been associated with human colon tumors [16]. Expression levels of Aurora-B [17] and Aurora-C [12] were elevated in several cancer cell lines relative to normal fibroblasts. Aurora-C is located on chromosome 19q13.2 to 13.4, a region associated with loss of heterozygosity in ovarian cancer [18] and pancreatic carcinomas [19]. Thus, the inhibition of one or more Aurora kinases might be a novel chemotherapeutic strategy against cancer [20]. Recently, several reports by research groups in pharmaceutical and biotechnology companies describe small molecules that target the ATP-binding domain of Aurora kinases, and have effects in human tumor cell lines [21-23].

Despite the importance of model organisms in understanding Aurora kinase function, the evolutionary relationships among these variants are unclear. Two previous phylogenetic analyses of Aurora kinases were incomplete because the contemporary complement of Aurora kinases was unavailable [4] or certain family members, namely the Aurora-C kinases, were excluded [1]. Here, we present an evolutionary analysis of all known Aurora kinases. We show that vertebrate Aurora kinases evolved through a series of gene duplication events from a chordate ancestor, and that they are highly distinct from invertebrate homologs. Moreover, the recent divergence, thus high level of sequence similarity, of human Aurora-B and Aurora-C suggests a novel anti-cancer strategy which might simultaneously target the ATP-binding domains of this kinase pair with dual action inhibitors.

## Results and discussion

### Aurora Evolution in Chordates

In order to construct a comprehensive phylogenetic tree, GenBank was searched for all possible Aurora kinases. In addition to previously published Aurora kinase sequences, further chordate and urochordate Aurora homologs were found by using mammalian Aurora-A, Aurora-B and Aurora-C protein sequences as queries in BLASTP or TBLASTN [24] searches of the genomes of the pufferfish, *Takifugu rubripes *[25], the zebrafish, *Danio rerio *[26], and the ascidian, *Ciona intestinalis *[27].

**Figure 1:**
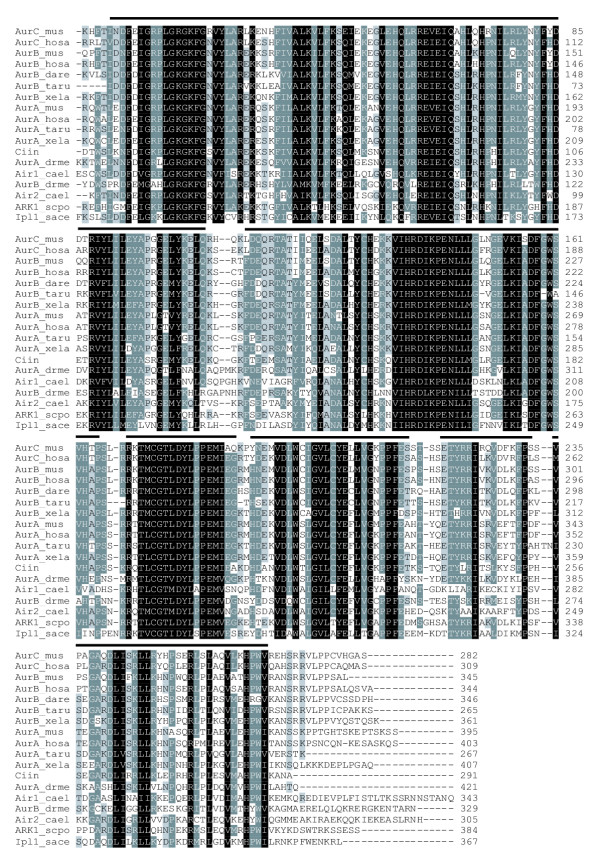
**Multiple sequence alignment of representative Aurora-A (AurA), Aurora-B (AurB), and Aurora-C (AurC) kinases, and their homologs (Air1, Air2, ARK1 and Ipl1). **N-terminal regions which are species-specific and could not be accurately aligned are excluded, although the numbering of residues begins at the starting amino acid for that particular peptide. Progressive darker shading indicates conservation of amino acid residues in 60%, 80% and 100% of the sequences, respectively. Dark line at the top of the sequence blocks indicates those regions used in the phylogenetic analyses (Also see additional file 1 and 2). Species include *Homo sapiens *(hosa), *Mus musculus *(mus), *Danio rerio *(dare), *Takifugu rubripes *(taru), *Xenopus laevis *(xela), *Ciona intestinalis *(ciin), *Drosophila melanogaster *(drme), *Caenorhabditis elegans *(cael), *Saccharomyces cerevisiae *(sace) and *Schizosaccharomyces pombe *(scpo). The program CLUSTALW [41] was used to constructed the initial alignment which was subsequently refined manually.

Multiple sequence alignments show that the Aurora kinase family is highly conserved among species (Fig. 1). Pairwise sequence comparisons estimate that the mean proportion of similar amino acids (based on the Blosum62 matrix) is much higher among all the different families of Aurora-A, Aurora-B and Aurora-C of vertebrates (0.84 ± 0.5) than within the same family (Aurora-A or Aurora-B) between vertebrates and invertebrates species (0.69 ± 0.3 for both families). This would suggest a recent evolutionary radiation of Aurora families within vertebrates.

**Figure 2:**
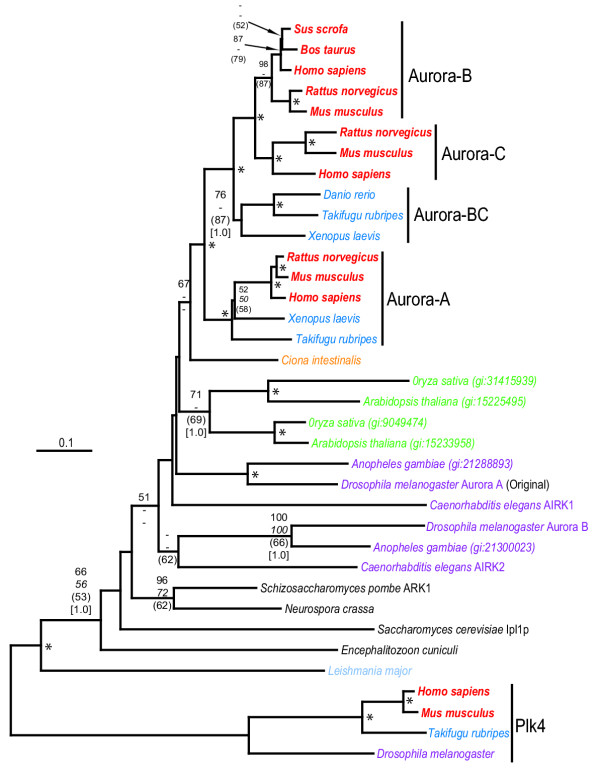
**Phylogenetic tree of Aurora-A, Aurora-B, and Aurora-C kinases rooted by PLK4 kinases. **Major organism groups (with colours, fonts) are mammals (red, bold italic), cold-blooded vertebrates (deep blue, italic), urochordates (orange, italic), invertebrates, (purple, italic), plants (green, italic), fungi (black, italic) and protists (light blue, italic). "Original" indicates the first Aurora identified from *Drosophila melanogaster *[3]. Plant sequences are identified by their Genbank accession number. Stacks of numbers show, in descending order, the percent occurrence of nodes in greater than 50% of 1000 bootstrap replicates of neighbor joining (plain text) and maximum parsimony (italicized text) analyses or greater than 50% of 10000 quartet puzzling steps of maximum likelihood analysis (in curved parentheses) or Bayesian posterior probability (only 0.90 or greater, in square parentheses). Asterisks ("*") indicate those nodes supported 70% or greater by the first three tree-building methods and 0.90 Bayesian posterior probability. Nodes with one or two values less than 50% have dashes ("-") while values less than 50% are unmarked. Scale bar represents 0.1 expected amino acid residue substitutions per site.

Phylogenetic trees constructed using four methodologies, all rooted using polo-like kinases type 4 (PLK4), show that all vertebrate Auroras form a clade distinct from those of invertebrates (Fig. 2). The phylogenetic tree constructed by the neighbour-joining distance method shows moderate boot-strap support (67%) for the evolution of all vertebrate Auroras from a urochordate ancestor, represented by the ascidian, *C. intestinalis*. The use of alternative kinase families, other than PLK4, to root the tree did not alter the internal topology of the Aurora clade. Although its genome sequence is incomplete, *C. intestinalis *likely has only a single Aurora homolog since other probable kinase open reading frames associated with the next top five BLASTP [24] hits did not cluster with Auroras from other species in phylogenetic trees.

Among true vertebrates, our phylogenetic tree shows that the Aurora kinases underwent two major gene duplication events. The first split in cold-blooded vertebrates lead to the formation of two Aurora subfamilies. One branch encompasses all known vertebrate Aurora-A sequences in a single orthologous lineage that includes fishes, amphibians and mammals. This family includes previously identified Aurora-A kinases in *Xenopus laevis*, rodents and humans as well as a new putative ortholog in *T. rubripes*.

The second family, previously known as Aurora-B [2,28] consists of cold-blooded vertebrate and mammalian Aurora-B as well as mammalian Aurora-C. Mammalian Aurora-B and Aurora-C are similarly related to the cold-blooded vertebrate Aurora presently known as "Aurora-B" in amphibians (*X. laevis*) and fish (*D. rerio *and *T. rubripes*). Searches of *T. rubripes *and *D. rerio *protein and DNA sequence databases detected several other putative serine/threonine kinase homologs but none were Auroras according to phylogenetic analyses. Thus, cold-blooded vertebrates appear to have only a single Aurora-A ortholog, a single Aurora-B-like homolog, and lack an Aurora-C ortholog. Conversely, Aurora-A, Aurora-B and Aurora-C appear to be ubiquitous to mammals (at least placentals) where they are encoded by separate chromosomal loci. It would appear that mammalian Aurora-B and Aurora-C evolved from a duplication event involving the ancestral Aurora-B found in cold-blooded vertebrates. This depiction of the evolutionary relationships of vertebrate Auroras was consistently determined by four different phylogenetic methods with high bootstrap or Bayesian posterior probability values (Fig. 2).

**Figure 3:**
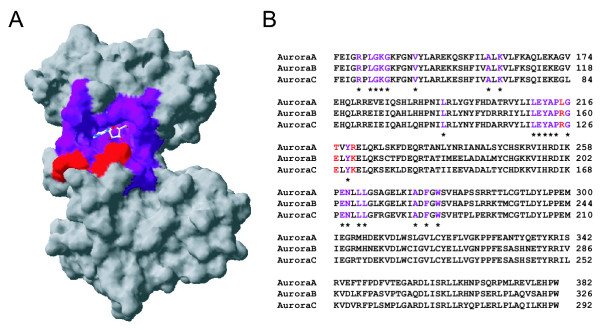
**Comparisons of the catalytic domains of human Aurora-A, Aurora-B and Aurora-C kinases. A. **Crystal structure of the catalytic domain of Human Aurora kinase with an adenosine molecule shown in the binding pocket (PDB ID 1muoA) [29]. Residues lining the active site are colored purple when invariant and red when variant. **B**, Multiple sequence alignment of Auroras. Using the same color scheme as the structure in panel A, residues identified to be lining the active site are identified with invariant residues among all three Auroras marked with an asterisk. Of the 26 residues lining the active site, only three vary among the different human Aurora kinases; Leu215, Thr217 and R220 (numbering and residue identity based on Aurora-A), and all of this variation was found in Aurora-A.

Comparisons of human Aurora-B and Aurora-C sequences to the resolved 3D structure of human Aurora-A [29] lends further support to the evolutionary scenario that vertebrate Aurora-B and Aurora-C are closely related paralogs (Fig. 3a). Of the 26 residues lining the ATP-binding active site, only three vary among the different human Aurora kinases; Leu215, Thr217 and R220 (numbering and residue identity based on Aurora-A), and all of these variants were specific to Aurora-A (Fig. 3b). Aurora-B and Aurora-C did not vary in their active site residues. Furthermore, all three Auroras have a carboxy-terminal destruction box (D-box) but only Aurora-A has the necessary amino-terminal A-box (also known as the D-box activating-domain) for its functional activation [30,31]. Collectively, these comparisons of structure and motifs support the phylogeny depicting an early divergence of Aurora-A from an Aurora-B / Aurora-C clade.

### Non-chordate Evolution

The Aurora kinases of plants and invertebrates are all outgroup lineages to chordates / urochordates (Fig. 2). Although all phylogenetic methods strongly support the monophyly of chordate Aurora kinases, the exact ordering among nodes leading to the various plant and invertebrate clusters were not resolved with similarly high bootstrap or probability values. Placement of plant Aurora kinases between chordates and invertebrates might be an artifact of tree construction methods. (Plant, protist, fungal and invertebrate lineages were all highly diverged from vertebrate Aurora kinases as witnessed by their longer branch lengths.) The earliest lineages of the Aurora tree are those fungal model organisms with a single Aurora-like homolog *S. cerevisiae *(Ipl1) and *S. pombe *(Ark1). Other basal branches are the amitochondrial fungi, *Encephalitozoon cuniculi*, and the kinetoplast protist, *Leishmania major *[32].

**Figure 4:**
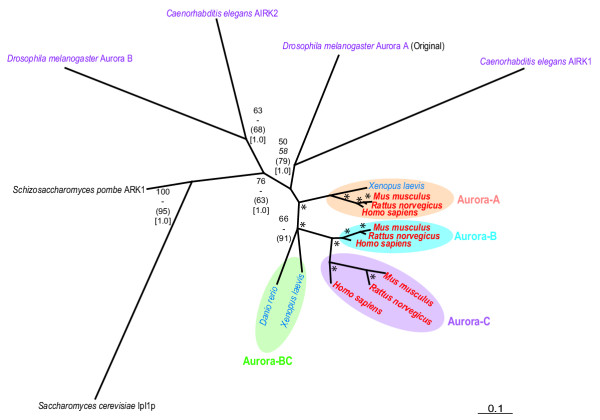
**Unrooted phylogenetic tree of Aurora kinases from human and model organisms. **Tree was constructed using the maximum likelihood quartet puzzling method [43]. Scale bar represents 0.1 expected amino acid residue substitutions per site. Confidence estimates of nodes, fonts, and colours of species names correspond to Fig. 2.

Invertebrate Aurora kinases, including those of the model organisms *C. elegans *and *D. melanogaster*, occupy separate early branches and are not, as their current names suggest, orthologs to either Aurora-A or Aurora-B of vertebrates. An unrooted phylogenetic tree with only model organism species shows the same topology of vertebrate Auroras as the more species-rich tree rooted by PLK4 kinases (Fig. 4). However, similar kinases from *C. elegans *and *D. melanogaster *now cluster together. The unrooted tree suggests that the invertebrate Aurora-B kinase family evolved prior to the invertebrate Aurora-A kinase family although further examples from other species are desirable to confirm this hypothesis. The consensus scenario in both rooted and unrooted trees is that vertebrate Aurora kinases are paralogous, rather than orthologous, to their invertebrate counterparts.

### Model Organisms in Context

Aurora-B and Aurora-C, as specific innovations in mammals, might have distinct protein-binding partners and cellular functions from those of Aurora-B kinases in amphibians. The perturbation of Aurora-B function in different systems suggests variable kinetochore-microtubule interactions [33]. Transfection of normal rat kidney cells with a kinase-inactive, dominant negative form of Aurora-B caused multiple defects in mitosis [34] while an Aurora-B kinase inactivating antibody seemed to have milder effects in *Xenopus *tissue culture cells [35]. *Xenopus *Aurora-A functions in the extrusion of the first polar body [36] while in *C. elegans *Aurora B plays a similar role [5]. Also, *C. elegans *Aurora-B binds to a protein CSC-1 which has no homolog in other studied systems [37]. While these studies used different experimental methods, the lack of direct orthology among vertebrate and invertebrate Aurora-A and Aurora-B might also account for functional differences in these systems.

The evolutionary analysis presented here also suggests revisiting the present Aurora nomenclature. Adams *et al. *[28] proposed a naming scheme where, irrespective of species, the original Aurora is known as Aurora-A (also called AIRK1, Aurora, Aurora-2, AIK, BTAK, human STK15, mouse STK6 and others), followed by Aurora-B (also known as AIRK-2, IAL, Aurora-1, AIK2, STK12 and others) and Aurora-C (or STK13). However, the proposed nomenclature fails to reflect evolutionary, and possibly functional, relationships among the Auroras. We suggest that Aurora-A be retained as the name for all orthologs in mammals and cold-blooded vertebrates. While Aurora-B and Aurora-C seem appropriate for mammalian versions, the ancestral cold-blooded vertebrate "Aurora-B" might be renamed "Aurora-BC". As for invertebrates, the so-called Aurora-A or Aurora-B genes are clearly not orthologs to their respective vertebrate counterparts. However, introducing a new nomenclature here might simply add further confusion to the field.

### Evolution of an Anti-Cancer Target

There have been several recent reports of Aurora kinase inhibitors that are under development by pharmaceutical or biotechnology companies for cancer treatment. The compounds Hesperadin (Boehringer Ingelheim [21]) and ZM447439 (AstraZeneca [22]) are suggested to be targeted to Aurora-B. While both studies show lesser levels of compound inhibition of Aurora-A as well as several other kinases, neither report included Aurora-C in their kinase profile. Selective inactivation of multiple kinases is not an undesirable pharmaceutical profile for a small molecule inhibitor and, in fact, could be the best strategy to achieve maximal clinical efficacy of an anti-cancer agent [38]. Indeed, an intense area of anti-cancer research is the development of small molecular ATP analogues that generally target the kinase domain of protein kinases [39]. For example, Gleevec (also known as imatinib and made by Novartis) for chronic myelogenous leukemia, is a small-molecule inhibitor that targets BCR-ABL, c-Kit and platelet-derived growth factor receptor kinases [40]. Recently, a selective inhibitor of all three Aurora kinases, VX-680 (made by Vertex Pharmaceuticals), was reported to inhibit cell-cycle progression and induce apoptosis in various human tumor cell types and in vivo xenograft models [23]. Interestingly, although VX-680 is a potent inhibitor of all three Aurora kinases, its apparent inhibition constant is much lower for Aurora-A (0.6 nM) than for either Aurora-B (18 nM) or Aurora-C (4.6 nM). Again, the compound's greater affinity for Aurora-A, relative to Aurora-B and Aurora-C is compatible with the proposed evolutionary scenario of mammalian Auroras.

## Conclusions

Evolutionary analysis shows that cell division Aurora kinases, while consistent in theme throughout eukaryotes, have undergone lineage-specific expansions and specialization in metazoans. Aurora-C is the least known of the Aurora kinases. Yet as an evolutionary innovation in mammals, further studies are very much warranted from the perspectives of better understanding its potential roles in both cell replication and tumor progression. A better functional understanding of Aurora-C would help clarify the evolutionary relationships of Aurora-B and Aurora-C in mammals relative to the ancestral Aurora-BC in cold-blooded vertebrates. Additionally, the close evolutionary and structural relationships between mammalian Aurora-B and Aurora-C offers the tantalizing opportunity to design dual kinase inhibitors that might circumvent potential tumor cell resistance to mono-target chemotherapeutics.

## Methods

### Database searches

All Aurora kinase orthologs and paralogs were initially collected from GenBank nonredundant protein database by performing separate searches using BLASTP [24] with human Aurora-A, Aurora-B and Aurora-C proteins as query sequences and a cut-off E-value of 1.0e-10. Since this dataset included additional kinases to the Auroras, preliminary multiple sequence alignments and phylogenetic analysis using program CLUSTALW v1.7 [41] served to identify the clade of all known Aurora kinases. *Takifugu rubripes*, *Danio rerios *and *Ciona intestinalis *homologs were obtained by BLASTP and TBLASTN [24] of species-specific protein and DNA sequence databases, respectively. The top five homologs from each species retrieved from separate searches with human Aurora-A, Aurora-B and Aurora-C were entered into a preliminary phylogenetic analysis using all retrieved Aurora kinases from Genbank. These analyses revealed that *T. rubripes *and *D. rerios *had orthologs to *X. laevis *Aurora-A and Aurora-B but not mammalian Aurora-C. *C. intestinalis *had a single Aurora-like kinase.

### Phylogenetic and structure analysis

PLK4 kinases were selected as the outgroup for phylogenetic analyses because they were the most similar non-Aurora kinases to either human Aurora-A, Aurora-B or Aurora-C in multiple BLASTP [24] searches of the non-redundant protein database of GenBank. Using alternative kinases as outgroups made no difference to the topology of the Aurora clade. Initial multiple sequence alignments were performed using the program CLUSTALW v1.7 [41] with default settings and subsequently, refined manually using the program SEQLAB of the GCG Wisconsin Package v11.0 software package (Accelrys, San Diego, CA, USA). We removed regions with residues that could not be unambiguously aligned or that contained insertions or deletions. The final multiple sequence alignment was 240 amino acids in length. Pairwise comparisons for the proportion of similar residues were estimated from the length of the shortest sequence without gaps and the Blosum62 weighting matrix as implemented in the program OLDDISTANCES in GCG.

We constructed phylogenetic trees using distance neighbor-joining (NJ), maximum parsimony (MP), maximum likelihood quartet puzzling (QP), and Bayesian posterior probabilities (BP). NJ trees were based on pair wise distances between amino acid sequences using the programs NEIGHBOR and PROTDIST (Dayhoff option) of the PHYLIP 3.6 package [42]. The programs SEQBOOT and CONSENSE were used to estimate the confidence limits of branching points from 1000 bootstrap replications. ML tree topologies were constructed using the software PUZZLE 4.0 [43], employing 1000 puzzling steps, the JTT substitution matrix, estimation of rate heterogeneity using the gamma distribution model with eight rate categories, and the gamma-parameter estimation from the dataset. MP analysis was performed using PAUP4.0b5 software [44] where the number and lengths of minimal trees were estimated from 100 random sequence additions, while confidence limits of branch points were estimated by 1000 bootstrap replications. BP trees were constructed using the software MrBayes v3.0B4 [45,46]. Bayesian analysis used the mixed model of sequence evolution with random starting trees. Markov chains were run for 10^6 ^generations, burn-in values were set for 10^4 ^generations, and trees sampled every 100 generations. All trees were visualized using the program TREEVIEW v1.6.6 [47].

For the Aurora kinase phylogeny rooted with PLK4 kinases shown in Fig. 2, the log likelihood of the final ML tree was -8059.78. Four minimal length MP trees were recovered, 1522 steps in length with a consistency index (CI) of 0.5802 and a retention index (RI) of 0.6016. The variable branch arrangements were terminal nodes (human, pig and cow Aurora-B) which did not affect the central findings.

For the unrooted phylogeny of Aurora kinases of model organisms shown in Fig. 3, the log likelihood of the final ML tree was -4469.20. A single minimal length MP trees were recovered, 779 steps in length with a consistency index (CI) of 0.7214 and a retention index (RI) of 0.2786.

The SwissPDBviewer program [48] was used to obtain the surface representation of human Aurora-A kinase (PDB ID 1muoA). The active site residues, defined as being within 5A of the ADP cofactor, were identified using the program CAST [49]. The multiple sequence alignment for the three human Aurora kinase proteins was obtained using CLUSTALW [41]. Multiple sequence alignment and sequence GenBank accession numbers are available as Supplementary Information [see additional file 1 and 2].

## Authors' contributions

JRB conceived the study, performed the phylogenetic analysis and drafted the manuscript. KKK performed the 3D structural analyses and contributed to the draft manuscript. MLB participated in the preliminary phylogenetic analysis and contributed to the draft manuscript. PS and DRP both contributed to the draft of the manuscript and added key references.

## Supplementary Material

Additional File 1 Multiple sequence alignment of edited Aurora and Plk4 kinases used to produce the phylogeny shown in Fig. 2. Click here for file

Additional File 2 Accession numbers (gi), Locus link Ids (LID) or genome predicted protein Ids for Aurora and PLK kinases as labelled in the Supplementary Data multiple sequence alignment file S1. Click here for file
