# Supplementary material for: Evolutionary relationships of Aurora kinases: Implications for model organism studies and the development of anti-cancer drugs
Source: BMC Evol Biol. 2004 Oct 12;4:39. doi: 10.1186/1471-2148-4-39 (PMC524484; doi:10.1186/1471-2148-4-39)
Supplement: Additional File 1 — Multiple sequence alignment of edited Aurora and Plk4 kinases used to produce the phylogeny shown in Fig. 2. [file 1471-2148-4-39-S1.doc]

S1. Multiple sequence alignment of edited Aurora and Plk4 kinases used to produce the phylogeny shown in Fig. 2.

* 20 * 40 * 60 * 80 *
AurC_rano : INDFEIGRPLGRGKFGRVYLARLKENHFIVALKVLFKSEIGKEGLEHQLRREVEIQSHLQHPNILRLYNYFYDDSRVYLILEYAPRGELYKELQLNQ : 97
AurC_mumu : INDFEIGRPLGRGKFGRVYLARLKENHFIVALKVLFKSEIEKEGLEHQLRREVEIQAHLQHRNILRLYNYFYDDTRIYLILEYAPGGELYKELQLDQ : 97
AurC_hosa : VDDFEIGRPLGKGKFGNVYLARLKESHFIVALKVLFKSQIEKEGLEHQLRREIEIQAHLQHPNILRLYNYFHDARRVYLILEYAPRGELYKELQLDE : 97
AurB_mumu : IDNFEIGRPLGKGKFGNVYLAREKKSRFIVALKILFKSQIEKEGVEHQLRREIEIQAHLKHPNILQLYNYFYDQQRIYLILEYAPRGELYKELQFDE : 97
AurB_rano : IDNFEIGRPLGKGKFGNVYLAREKKSRFIVALKILFKSQIEKEGVEHQLRREIEIQAHLKHPNILQLYNYFYDQQRIYLILEYAPRGELYKELQFDE : 97
AurB_susc : IDDFEIGRPLGKGKFGNVYLAREKKSHFIVALKVLFKSQIEKEGVEHQLRREIEIQAHLQHPNILRLYNYFYDRRRIYLILEYAPRXELYKELQFDE : 97
AurB_bota : IDDFEIGRPLGKGKFGNVYLAREKKSHFIVALKVLFKSQIEKEGVEHQLRREIEIQAHLQHPNILRLYNYFYDRRRIYLILEYAPRGELYKELQFDE : 97
AurB_hosa : IDDFEIGRPLGKGKFGNVYLAREKKSHFIVALKVLFKSQIEKEGVEHQLRREIEIQAHLHHPNILRLYNYFYDRRRIYLILEYAPRGELYKELQFDE : 97
AurBC_dare : IDDFDIGRPLGKGKFGNVYLARERKLKVVIALKVLFKSQMVKEGVEHQLRREIEIQSHLRHPNILRFYNYFHDDTRVFLILEYAPRGEMYKELQFDD : 97
AurBC_tafu : IDDFDIGRPLGKGKFGNVYLARVKKLEAIVALKVLFKSQMEKEGVEHQLRREIEIQSHLKHPNILRFYNYFHDRKRVFLVLEYAPRGEMYKELQFDD : 97
AurBC_xela : IDDFDIGRPLGKGKFGNVYLAREKQNKFIMALKVLFKSQLEKEGVEHQLRREIEIQSHLRHPNILRMYNYFHDRKRIYLMLEFAPRGELYKELQFDE : 97
AurA_rano : LEDFDIGRPLGKGKFGNVYLAREKQSKFILALKVLFKVQLEKAGVEHQLRREVEIQSHLRHPNILRLYGYFHDATRVYLILEYAPLGTVYRELQFDE : 97
AurA_mumu : LEDFDIGRPLGKGKFGNVYLARERQSKFILALKVLFKTQLEKANVEHQLRREVEIQSHLRHPNILRLYGYFHDATRVYLILEYAPLGTVYRELQFDE : 97
AurA_hosa : LEDFEIGRPLGKGKFGNVYLAREKQSKFILALKVLFKAQLEKAGVEHQLRREVEIQSHLRHPNILRLYGYFHDATRVYLILEYAPLGTVYRELQFDE : 97
AurA_tafu : LENFDIGRPLGKGKFGNVYLARERQSRFILALKVLFKKQLEKAGVEHQLRREVEIQSHLRHPNILRLYGYFHDPSRVYLILEFAPKGELYGELQFPE : 97
AurA_xela : LEDFEIGRPLGKGKFGNVYLARERESKFILALKVLFKSQLEKAGVEHQLRREVEIQSHLRHPNILRLYGYFHDASRVYLILDYAPGGELFRELQFDD : 97
ciin : LKNFDIGKPLGRGKFGSVYLAREKKSKFIVALKVLFKSQLMKSNVEHQLRREIEIQSHLRHPHILRLYGYFHDETRVYLILEYASRGEMYKELQFTE : 97
arth152339 : LSDFDIGKPLGRGKFGHVYLAREKRSNHVVALKVLFKSQLQQSQVEHQLRREVEIQSHLRHPNILRLYGYFYDQKRVYLILEYAARGELYKDLQFSE : 97
orsa904947 : LSDFDIGKPLGRGKFGHVYLAREKRSNHIVALKVLFKSQLKQSQVEHQLRREVEIQSHLRHPNILRLYGYFYDT-RVYLILEYALKGELYKELQFSE : 96
arth152254 : LADFEIGRPLGKGKFGRVYLAREAKSKYIVALKVIFKEQIEKYKIHHQLRREMEIQTSLRHPNILRLFGWFHDNERIFLILEYAHGGELYGVLKLTE : 97
orsa314159 : MDDFEIGKYIGEGKFGKVYLAREKQSGYVVALKVTFKAKLDKYRFHAHLRREIEIQHGLDHPNVLRLFAWFHDAERVVLVLEYAARGELYKLLRFSE : 97
anga212888 : LSNFDIGRPLGRGKFGNVYLAREKETKFVIALKVLFKKQVHAQGIEHQVRREIEIQSHLRHPNILRMYGYFHDESRIYLILEYAPGGTLFKEQQFPE : 97
AIRK1_drme : LNNFDIGRLLGRGKFGNVYLAREKESQFVVALKVLFKRQIGESNVEHQVRREIEIQSHLRHPHILRLYAYFHDDVRIYLILEYAPQGTLFNALQFDE : 97
encu : LDRFEIGRLLGRGKFGQVWLAREREKGFIVALKIIPIKEIQTVETARQIRREIEIHSNLKHPNILRMYGHFHDKDNIYLILEYAGKGEFFKFLSFGE : 97
AIRK1_cael : LDDFDVGRPLGKGKFGNVFISREKKTKRIIALKVLFKTQLLQLGVSHQLKREIEIQYHLRHPNILTLYGYFHDDKRVFVILDYASRGELFNVLQVNE : 97
anga213000 : TDDFEVGRALGRGKFGRVYLARERETGFMVAMKVMFKSQLTKWHVEKQLLREIEIQSRLKHPHILRLYTWFHDDRRIYLALELAAQGELYKHLKFDE : 97
AIRK2_drme : PRDFEMGAHLGRGKFGRVYLARERHSHYLVAMKVMFKEELRKGCVQRQVLREIEIQSRLKHPHILRLLTWFHDESRIYLALEIASEGELFKHLRFDE : 97
AIRK2_cael : INDFEIGRPLGKGKFGSVYLARTKTGHFHVAIKVLFKSQLISGGVEHQLEREIEIQSHLNHPNIIKLYTYFWDAKKIYLVLEYAPGGEMYKQLTFSE : 97
Ark1_scpo : IGMFEIGKPLGKGKFGRVYLAKEKKTGFIVALKTLHKSELVQSKIEKQVRREIEIQSNLRHKNILRLYGHFHDEKRIYLILEFAGRGELYQHLRFSE : 97
necr : LGMFEIGRPLGKGKFGRVYLARERSSGFICALKVLYKSELQHGGVEKQVRREIEIQSNLRHPNILKLYGHFHDSKRIFLILEYAGKGELYKHLRFPE : 97
Ipl1p_sace : LDDFELGKKLGKGKFGKVYCVRHRSTGYICALKVMEKEEIIKYNLQKQFRREVEIQTSLNHPNLTKSYGYFHDEKRVYLLMEYLVNGEMYKLLRFND : 97
lema : IHDFELLHKLGGGNYGDVYLASVRKSNYVVAIKKLSIKKLAEFDIVNQLRREIEIAFNTRHKYLLRTYAYFFDEHDIYLILEPCSNGMLYSELNFPP : 97
Plk4_hosa : IEDFKVGNLLGKGSFAGVYRAESIHSGLEVAIKMIDKKAMYKAGMVQRVQNEVKIHCQLKHPSILELYNYFEDSNYVYLVLEMCHNGEMNRYLKFSE : 97
Plk4_mumu : IEDFKVGNLLGKGSFAGVYRAESIHTGLEVAIKMIDKKAMYKAGMVQRVQNEVKIHCQLKHPSVLELYNYFEDNNYVYLVLEMCHNGEMNRYLKFSE : 97
Plk4_tafu : --DFKVLMLLGKGSFACVYRAKSVKTGLEVAIKTIDKKSMHKAGMVQRVTNEVEIQCRLKHPSILELYNYFEDSNYVYLVLEMCHNGEMSRYLKFSE : 95
PLK4_drme : IEDYEVQHLLGKGGFATVYKARCLHTHQDVAIKMIDKKLIQGTGLTNRVRQEVEIHSRLKHPSVLQLYTFFQDANYVYLVLELAHNGELHRYMNFTE : 97

100 * 120 * 140 * 160 * 180 *
AurC_rano : QRTATIIEELSDALIYCHRNKVIHRDLKPENLLLGLKGEVKIADFGWSVHTPRKTMCGTLDYLPPEMIEGYNETVDLWCIGVLCYELLVGKPPFESE : 194
AurC_mumu : QRTATIIQELSDALTYCHEKKVIHRDIKPENLLLGLNGEVKISDFGWSVHTPRKTMCGTLDYLPPEMIAQYNEMVDLWCIGVLCYELLVGKPPFESE : 194
AurC_hosa : QRTATIIEELADALTYCHDKKVIHRDIKPENLLLGFRGEVKIADFGWSVHTPRKTMCGTLDYLPPEMIEGYDEKVDLWCIGVLCYELLVGYPPFESE : 194
AurB_mumu : QRTATIMEELSDALTYCHKKKVIHRDIKPENLLLGLQGELKIADFGWSVHAPRKTMCGTLDYLPPEMIEGHNEMVDLWCIGVLCYELMVGNPPFESE : 194
AurB_rano : QRTATIMEELSDALMYCHKKKVIHRDIKPENLLLGLQGELKIADFGWSVHAPRKTMCGTLDYLPPEMIEGHNEMVDLWCIGVLCYELMVGNPPFESE : 194
AurB_susc : QRTATIMEELADALIYCHGKKVIHRDIKPENLLLGLQGELKIADFGWSVHAPRKTMCGTLDYLPPEMIEGHNEKVDLWCIGVLCYELLVGNPPFESE : 194
AurB_bota : QRTATIMEELADALTYCHAKKVIHRDIKPENLLLGLRGELKIADFGWSVHAPRKTMCGTLDYLPPEMIEGHNEKVDLWCIGVLCYELLVGNPPFESE : 194
AurB_hosa : QRTATIMEELADALMYCHGKKVIHRDIKPENLLLGLKGELKIADFGWSVHAPRKTMCGTLDYLPPEMIEGHNEKVDLWCIGVLCYELLVGNPPFESE : 194
AurBC_dare : QRTATYMEEVSDALQYCHEKKVIHRDIKPENLLLGYRGELKIADFGWSVHAPRRTMCGTLDYLPPEMIEGHDEKVDLWSIGVLCYECLVGNPPFETE : 194
AurBC_tafu : QRTATYMEEISDALLYCHERKVIHRDIKPENLLLG-VGELKIADF-WAVHAPRRTMCGTLDYLPPEMIEGHSEKVDLWCIGVLCYECLVGNPPFETE : 192
AurBC_xela : QRSATFMEELADALHYCHERKVIHRDIKPENLLMGYKGELKIADFGWSVHAPRRTMCGTLDYLPPEMIEGHDEKVDLWCAGVLCYEFLVGMPPFDSE : 194
AurA_rano : QRTATYITELANALSYCHSKRVIHRDIKPENLLLGSNGELKIADFGWSVHAPRTTLCGTLDYQPPEMIEGHDEKVDLWSLGVLCYEFLVGMPPFEAE : 194
AurA_mumu : QRTATYITELANTLSYCHSKRVIHRDIKPENLLLGSNGELKIADFGWSVHAPRTTMCGTLDYLPPEMIEGHDEKVDLWSLGVLCYEFLVGMPPFEAE : 194
AurA_hosa : QRTATYITELANALSYCHSKRVIHRDIKPENLLLGSAGELKIADFGWSVHAPRTTLCGTLDYLPPEMIEGHDEKVDLWSLGVLCYEFLVGKPPFEAE : 194
AurA_tafu : ERSATYIMELADALNYCHSKKVIHRDIKPENLLLGANGELKIADFGWSVHTPRSTLCGTLDYLPPEMIEGHDEKVDLWSLGVLCYEFLVGKPPFEAE : 194
AurA_xela : QRSAMYIKQLAEALLYCHSKKVIHRDIKPENLLLGSNGELKIADFGWSVHAPRTTLCGTLDYLPPEMIEGHDETVDLWSLGVLCYEFLVGKPPFETE : 194
ciin : EMSATYIAELADALNYCHSKQVIHRDIKPENLLMGLRGELKIADFGWSVHAPRQTLCGTLDYLPPEMIEAHDANVDLWTLGILCYEFLVGKPPFETE : 194
arth152339 : RRAATYVASLARALIYCHGKHVIHRDIKPENLLIGAQGELKIADFGWSVHTFRRTMCGTLDYLPPEMVESHDASVDIWSLGILCYEFLYGVPPFEAD : 194
orsa904947 : RRSATYIASLAHALIYLHGKHVIHRDIKPENLLIGSQGELKIADFGWSVHTFRRTMCGTLDYLPPEMVEKHDYHVDIWSLGILCYEFLYGVPPFEAE : 193
arth152254 : QQAATYIASLSQALAYCHGKCVIHRDIKPENLLLDHEGRLKIADFGWSVQSSRKTMCGTLDYLAPEMVENHDYAVDNWTLGILCYEFLYGNPPFEAD : 194
orsa314159 : RTAATYVASLAGALAYCHKKQVIHRDIKPENLLLDIEGRLKIADFGWAVRSNRHTLCGTIDYLAPEMIEKHDHAVDNWTLGILCYEFLYGSPPFEAD : 194
anga212888 : KRCAIYVYSLVSALIYLHERNVIHRDIKPENLLLGHGGELKIADFGWSVHEPRTTLCGTLDYLSPEMVQGHTKTVDLWSLGVLAYELLCGKAPFLAE : 194
AIRK1_drme : RQSATYIQALCSALLYLHERDIIHRDIKPENLLLGHKGVLKIADFGWSVHEPRMTLCGTVDYLPPEMVQGHTKNVDLWSLGVLCFELLVGHAPFYSE : 194
encu : KETSLYIRQVMLALTYMKECNVIHRDIKPENLLLGSDNQLKIADFGWAVYNARMTFCGTMEYLAPEMVNNHDSGIDLWCLGILTYEFLMGKTPFESE : 194
AIRK1_cael : VIAGRFVRQLANALHYCHSKGVIHRDIKPENLLLDSKLNLKLADFGWSVVADRHTLCGTMDYLAPEMVSNHDFNVDIWAIGILLFEMLVGYAPFANK : 194
anga213000 : RRSARYISQVADALNYCHANNVIHRDLKPENILLTDEDNIKLADFGWSAHTNRKTMCGTLDYLPPEMVDGYDDSVDQWCLGILCYEFLVGNPPFEST : 194
AIRK2_drme : PRSAKYTYQVANALNYCHLNNVIHRDLKPENILLTSTDDLKLADFGWSAHTPRRTLCGTLDYLPPEMVDGYDDSVDQWCLGILCYEFVVGCPPFESS : 194
AIRK2_cael : PTAAKYMYEIADALSYCHRKNVIHRDIKPENLLIGSQGELKIGDFGWSVHAPRQTMCGTMDYLPPEMVNGHSDAVDLWAIGVLCYEFLVGKPPFEHK : 194
Ark1_scpo : EVASKYIFQMANALSYLHKKHVIHRDIKPENILLGIDGEIKLSDFGWSVHAPRTTLCGTLDYLPPEMVEGHTEKVDLWSLGVLTYEFLVGAPPFEDA : 194
necr : WKAAQYVAQMASALKYLHRKHVIHRDIKPENILVGIHGEIKISDFGWSVHAPRQTLCGTLDYLPPEMIRSYNEKVDLWSLGVLTYEFLVGEAPFEDM : 194
Ipl1p_sace : ILASDYIYQIANALDYMHKKNIIHRDIKPENILIGFNNVIKLTDFGWSIINPRKTVCGTIDYLSPEMVESYDHTIDAWALGVLAFELLTGAPPFEET : 194
lema : PTAARYVAQLAEALLYLHQHHILHRDIKPENILLDHHQNIKLADFGWSVHDPRKTSCGTPEYFPPEIVSRYDMSADLWCLGIFCFELLVGHTPFVSQ : 194
Plk4_hosa : NEARHFMHQIITGMLYLHSHGILHRDLTLSNLLLTRNMNIKIADFGLATQLKHYTLCGTPNYISPEIATRHGLESDVWSLGCMFYTLLIGRPPFDTN : 194
Plk4_mumu : REARHFMHQIITGMLYLHSHGILHRDLTLSNILLTRNMNIKIADFGLATQLNHYTLCGTPNYISPEIATRHGLESDIWSLGCMFYTLLIGRPPFDTN : 194
Plk4_tafu : DEARHFMHQIVKGMLYLHTHGILHRDLTLSNLLLTNNMNIKIADFGLATQLKHFTMCGTPNYISPEVATRHGLESDVWSLGCMFYAFLMGRPPFDTH : 192
PLK4_drme : TEAASILKQVVAGLLYLHSHNIMHRDISLSNLLLSREMHVKIADFGLATQLKHMTMCGTPNYISPEVVSRHGLPADVWSVGCMLYTLLVGRPPFETS : 194

200 * 220 * 240
AurC_rano : TCRRICQVDFRFPSMPAGAQDLISKLLRHHPSERLSLAQVLKHPWV : 240
AurC_mumu : TYRRIRQVDFKFPSVPAGAQDLISKLLRYHPSERLSLAQVLKHPWV : 240
AurC_hosa : TYRRILKVDVRFPLMPLGARDLISRLLRYQPLERLPLAQILKHPWV : 240
AurB_mumu : TYRRIVKVDLKFPSVPSGAQDLIFKLLKHNPWQRLPLAEVATHPWV : 240
AurB_rano : TYRRIVKVDLKFPSMPLGAKDLISKLLKHNPSQRLPLEQVSAHPWV : 240
AurB_susc : TYRRIGKVDLKFPPVPAGAQDLISKLLKHNPSDRLPLAQVSAHPWV : 240
AurB_bota : TYRRIVKVDLKFPPVPLGAQDFIYKLLKHNPSERLPLAQVSAHPWV : 240
AurB_hosa : TYRRIVKVDLKFPAVPTGAQDLISKLLRHNPSERLPLAQVSAHPWV : 240
AurBC_dare : TYKRITKVDLQFPKVSEGARDLISKLLRHSPSMRLPLRSVMEHRGV : 240
AurBC_tafu : TYKRITKVDLKFPKVSDGARDLISKLLRHNPIDRLTLQNVIDHPWV : 238
AurBC_xela : THRRIVNVDLKFPPLSDGSKDLISKLLRYHPPQRLPLKGVMEHPWV : 240
AurA_rano : TYRRISRVEFTFPDVTEGARDLISRLLKHNSSQRLTLAEVLEHPWI : 240
AurA_mumu : TYRRISRVEFTFPDVTEGARDLISRLLKHNASQRLTLAEVLEHPWI : 240
AurA_hosa : TYKRISRVEFTFPDVTEGARDLISRLLKHNPSQRPMLREVLEHPWI : 240
AurA_tafu : TYRRISRVEYTYPAISDGAKDLVSRLLKHNPMQRLPVQGVLAHPWV : 240
AurA_xela : TYRRISKVEFQYPPVSEEARDLVSKLLKHNPNHRLPLKGVLEHPWI : 240
ciin : TYLRITSLKYSFPPVSEGARDLIRRLLKLEPRHRLPLESVMAHPWI : 240
arth152339 : TYRRIVQVDLKFPPISASAKDLISQMLVKESSQRLPLHKLLEHPWI : 240
orsa904947 : TYRRIVKVDLKFPLVSPAAKDLISQMLVKNSAHRLPLHKLLEHPWI : 239
arth152254 : TFKRILKIDLSFPLVSEEAKNLISQLLVKDPSKRLSIEKIMQHPWI : 240
orsa314159 : TLRRIVKVDLSFPSVSADAKDLICKLLVKDSNKRLSLDDIMKHPWI : 240
anga212888 : TYRKIMKVQYTVPPVTKAASHLISRLLVKDPASRMPLENVAIHPWI : 240
AIRK1_drme : TYKKILKVDYKLPEISKAASHLISKLLVLNPQHRLPLDQVMVHPWI : 240
encu : AYKKINSLKYTIPEISSNASDFISRLLVLSPGDRMELTEALNHPFI : 240
AIRK1_cael : LIARIKECKIYIPSVTDGAASLINAIIKKEPQERLPLVDIMAHPWI : 240
anga213000 : TYDKIRRLDIVYPRMTAGAINLISKLLRIPSSSRITLRDVMNHPWV : 240
AIRK2_drme : TYSKIRRMEISYPSLSKGCKELIGGLLRKESKGRITLVDVMTHYWV : 240
AIRK2_cael : TYAAIKAARFTYPDVKKGARDLIGRLLVVDPKARCTLEQVKEHYWI : 240
Ark1_scpo : TYKRIAKVDLKIPSVPPDARDLISRLLQHNPEKRMSLEQVMRHPWI : 240
necr : TQKRIARADMTIPEVSKEAKDLIKKLLVLDPEKRLPLEEVENHPWI : 240
Ipl1p_sace : TYKRIAALDIKMPSISQDAQDLILKLLKYDPKDRMRLGDVKMHPWI : 240
lema : IYKKIHAMQYTIPDVPPEAKDLISNLLIREGSKRLALHRVLSHPFL : 240
Plk4_hosa : TLNKVVLADYEMPTLSIEAKDLIHQLLRRNPADRLSLSSVLDHPFM : 240
Plk4_mumu : TLNKVVLADYEMPALSREAQDLIHQLLRRNPADRLSLSSVLDHPFM : 240
Plk4_tafu : TLSKVVLGEYEMPAASLEAQDLIHQLLQKDPAQRPSLSAVLDHPFM : 238
PLK4_drme : TLNKVVMSEYIMPALSYEAQDLINKLLKKLPHERITLEAVLCHPFM : 240
